# The shape of the systolic blood pressure response during graded exercise: methodology, correlates and predictive value

**DOI:** 10.1038/s41371-025-01093-7

**Published:** 2025-11-14

**Authors:** Nicholas Cauwenberghs, Anna Carlén, Thomas Lindow, Viktor Elmberg, Lars Brudin, Magnus Ekström, Kristofer Hedman

**Affiliations:** 1 Research Unit Hypertension and Cardiovascular Epidemiology, Department of Cardiovascular Sciences, KU Leuven, Leuven, Belgium; 2 Department of Clinical Physiology in Linköping, and Department of Health, Medicine and Caring Sciences, Linköping University, Linköping, Sweden; 3 Department of Physical Education and Sports, Sport and Health University Research Institute (iMUDS), University of Granada, Granada, Spain; 4 Department of Medicine, Department of Research and Development, Växjö Central Hospital, Region Kronoberg, and Clinical Sciences, Pulmonary Medicine, Allergology and Palliative Medicine, Lund University, Lund, Sweden; 5 Lund University, Faculty of Medicine, Department of Clinical Sciences Lund, Respiratory Medicine and Allergology, Lund, Sweden; 6 Department of Clinical Physiology, Blekinge Hospital, Karlskrona, Sweden; 7 Department of Clinical Physiology, Kalmar County Hospital, Kalmar, Sweden

**Keywords:** Hypertension, Prognosis

## Abstract

During graded exercise, systolic blood pressure (SBP) is expected to increase linearly, but other responses are observed. To date, a framework for algorithmic assessment of the shape of the SBP response is lacking, making its physiological and clinical relevance poorly understood. We aimed to algorithmically identify distinct SBP response shapes and analyze their association with clinical factors and all-cause mortality. We retrospectively analyzed SBP recordings from a cohort of 5633 patients (mean age, 55.1 years; 43% female) undergoing maximal cycle ergometry, who met strict quality criteria, including ≥30 mmHg increase in SBP during exercise. Per patient, test duration and SBP values were rescaled (0-100%) to retrieve the SBP response shape. Group-based trajectory modelling (GBTM) was used to classify SBP shapes by sex. Associations with clinical factors and all-cause mortality were evaluated using multinomial logistic regression and Cox survival analysis. In both sexes, GBTM identified three SBP response shapes: early, linear and late rise in SBP. Late rise was associated with higher resting SBP, lower peak SBP and smaller increases in SBP during exercise (P < 0.05). A late SBP rise related independently to older age, higher body mass index, beta blocker use and lower exercise capacity. A late SBP rise predicted increased all-cause mortality in men (HR_adjusted_ versus early SBP rise: 1.66, 95% CI: 1.10-2.50; P = 0.015) but not in women (1.06, 0.60-1.90; *P* = 0.84). In conclusion, a late SBP response was linked to a worse risk profile and independently predicted all-cause mortality in men, suggesting clinical relevance for SBP shape assessment during exercise.

## Introduction

Systolic blood pressure (SBP) is monitored routinely during clinical exercise tests as it provides insights into cardiovascular (CV) function, hemodynamic response, and overall CV health [1]. Under normal physiological conditions, SBP is expected to rise linearly with increasing workload, following the increase in cardiac output required to meet the body’s higher metabolic demand [2]. Both abnormally high and low peak SBP have been linked to adverse outcomes [3–7]. For instance, a drop in SBP (typically >10 mmHg), is a well-established adverse prognostic marker associated with increased risk of all-cause and CV mortality [3]. Additionally, a failure to increase SBP may indicate a clinically significant heart disease such as severe left ventricular systolic dysfunction and ischemic heart disease, regardless of whether the SBP drop occurs immediately or after an initial increase [8]. Conversely, an exaggerated SBP response may indicate an increased risk of masked and future hypertension and CV events [4–7, 9].

Previous studies extracted discrete prognostic markers from the SBP response to exercise by investigating peak SBP values [10] or from straight SBP slopes between two time points [11, 12], with or without adjusting for exercise capacity. However, for a deeper understanding of the SBP response to exercise, we may need to consider the entire SBP trajectory, from start to peak exercise. As such, evaluating the manner in which peak SBP is reached may provide physiologically and clinically relevant information beyond discrete metrics like peak SBP. In clinic settings, other response shapes than linear have been observed, even in patients with an adequate rise in SBP during exercise. Yet, these non-linear trajectories have not yet been systematically described, as there is currently no objective way to evaluate the (non-)linearity of an SBP response shape. As a result, the physiological and clinical relevance of the SBP response shape to exercise is poorly understood.

To address this gap, trajectory modelling techniques may help to objectively analyze SBP response shapes during graded exercise. These algorithmic approaches enable unbiased characterization of time-series data [13, 14], potentially allowing for the identification of distinct SBP response groups (e.g. early vs. late rise). However, the concept of SBP shape characterization remains unexplored. Therefore, using data of a large retrospective cohort of patients referred for graded cycle ergometry, we aimed to algorithmically identify distinct SBP response shapes and assess their associations with clinical factors and all-cause mortality.

## Methods

### Study cohort

From a retrospective database of clinical graded exercise tests from 14,401 patients [15, 16], we excluded non-cycle ergometer tests (n = 643), tests in minors (n = 622) and submaximal tests defined as a rate of perceived exertion below 17 or missing (n = 1234) or as a test under 6 min (n = 2036). We additionally excluded 3594 patients whose SBP recording did not met the strict quality criteria to assess the SBP shape reliably, e.g. patients with less than 4 SBP measurements during exercise or with the first exercise SBP measured after more than 200 seconds (Supplemental Fig. 1). As we aimed to identify SBP shapes in patients with a meaningful rise in SBP, patients who failed to increase their SBP during exercise by 30 mmHg or more were excluded during this step (n = 569). Furthermore, we excluded patients with recorded diagnosis of valvular heart disease (n = 120), a pacemaker (n = 1) and, due to a high risk for inaccurate SBP measurements, morbid obesity (n = 149) or arrhythmia during the test (n = 361). Additionally, 8 people were excluded due to insufficient quality of the SBP recording noticed when pre-processing the SBP recordings for analysis, bringing the total analyzable sample to 5633 patients (Supplemental Fig. 1). For sensitivity analyses, we identified a subsample of 2893 apparently healthy individuals, free from traditional CV risk factors (i.e. hypertension, diabetes mellitus, and obesity) and CV disease, chronic obstructive pulmonary disease (COPD) and renal failure.

### Exercise testing

All graded exercise tests were performed on an electrically braked bicycle (Rodby Inc, Karlskoga, Sweden) [16]. Ramp protocols were applied, most starting at 30 Watts in women and 50 Watts in men, followed by increases in workload of 10 Watts/min in women and 15 Watts/min in men until maximal volitional exertion. As clinically recommended, a total exercise time of 8 to 12 min was aimed for. SBP was measured in the right arm using a Doppler probe over the radial artery, with manual cuff inflation/deflation, before the start of exercise (resting SBP) and every 2–3 min during cycling (exercise SBP). The peak work rate was recorded and the percentage predicted workload (ppWL) was calculated from the patient’s age, sex and height [17]. Afterwards, temporal data was exported, including the individual, time-stamped SBP measurements recorded during the exercise test.

### Medical data

The exercise data were crosslinked to mandatory Swedish national registries covering in- and outpatient diagnoses, dispensed outpatient medications and mortality as detailed elsewhere [15, 16]. Cause and date of death were determined through the Swedish Causes of Death register (up until 30 April 2019). The completeness and reliability of this registry is well established [18].

### Data management and statistical analysis

**Fig. 1:**
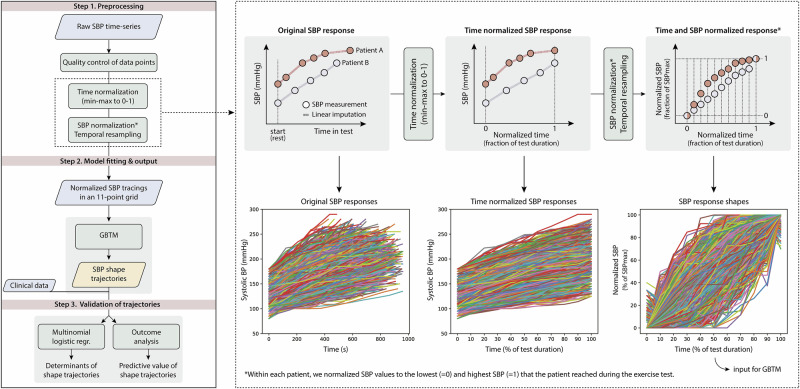
Analytical workflow to derive and validate groups based on the shape of the SBP response during graded exercise. Rhomboids represent input and output data. Rectangles indicate analytical steps. Abbreviations: GBTM group-based trajectory modelling, SBP systolic blood pressure.

Database management and statistical analyses were conducted within a Python 3.14 environment using common data science libraries such as pandas, statsmodels and matplotlib. Figure 1 presents the 3-step analytical workflow applied to identify and validate phenogroups based on the shape of the SBP response during graded exercise.

*Step 1: Preprocessing of SBP time-series*. First, large drops ( > 20 mmHg) or increases (>60 mmHg) between consecutive data points were flagged and visually reviewed. Implausible values, likely reflecting measurement errors or typos, were removed, while physiologically consistent changes in the SBP recording were retained. In case the resting sitting SBP exceeded the first exercise value by >10 mmHg, the resting SBP was adjusted to the first exercise SBP + 10 mmHg. This step aimed to reduce the impact of anticipatory effects (e.g. due to nervousness prior to the test), which else cause artefactual drops at the beginning of the SBP response that would disproportionally influence trajectory modelling. Within each patient, the test duration and SBP values were rescaled from 0 to 100% using min-max normalization (Fig. 1). Normalization of the SBP values yielded the SBP response shape, while normalization of the test time enabled comparison of SBP response shapes across individuals with different test durations. Normalizing to workload instead of test time is equally valid in this context, as workload increased incrementally. Time and workload thus increased proportionally throughout the test. Both approaches yield practically identical SBP response shapes, as observed by near perfect within-patient correlations between the normalized time and workload axes (r = 0.99 ± 0.006). Temporal resampling via linear interpolation produced evenly spaced 11-point SBP shape tracings, providing one data point per 10% of test duration (Fig. 1).

*Step 2: Group-based trajectory modelling to classify SBP response shapes*. GBTM is a latent class approach used to identify distinct trajectories within a set of time-series [13]. Here, we applied GBTM to classify the cohort into discrete SBP shape response groups based on their SBP shape tracing. This analysis was conducted using the R package ‘gbmt’ (v0.1.3) [14] with the 0-1 normalized test duration as the time scale. The GBTM algorithm modelled 2 to 10 SBP response shapes in quadratic form using a maximum likelihood function [13]. In both men and women, the Bayesian information criterion (BIC) indicated that the 3-trajectory model provided the best fit (Supplemental Fig. 2). This model was subsequently used to classify the SBP response shapes. The three SBP response shapes provided by the 3-trajectory model were qualitatively labelled based on their visual appearance: ‘early rise’, ‘linear rise’ and ‘late rise’ in SBP. Lastly, each patient was assigned to the SBP response shape for which they had the greatest posterior predictive probability.

*Step 3: Validation of shape-based SBP response groups*. To evaluate the clinical relevance of the SBP response groups, we analyzed their associations with clinical factors and all-cause mortality.

First, clinical characteristics and discrete SBP metrics (e.g. peak SBP) were compared across the SBP response groups using pairwise Z-tests. In multinomial logistic regression, we assessed the multivariable-adjusted odds of belonging to each SBP response group relative to the ‘early rise’ group. Covariates included age, BMI, resting heart rate, hypertension, diabetes mellitus, renal failure, COPD, cardiac disease, the use of antihypertensive medication, beta blockers, lipid-lowering drugs, and anticoagulant drugs, and ppWL.

To evaluate the association between SBP response groups and all-cause mortality, Kaplan-Meier survival curves were plotted for each SBP response group with pairwise log-rank tests to compare survival distributions. Cox proportional hazards models were used to calculate hazard ratios (HR) for all-cause mortality, comparing each SBP response group to the “early rise” SBP response group. Adjusted HRs accounted for differences in age, BMI, resting SBP and heart rate, diabetes mellitus, renal failure, COPD, cardiac disease, antihypertensive drugs, beta blockers, lipid-lowering drugs, anticoagulant drugs and ppWL.

## Results

### Characterization of shape-based SBP response groups

**Fig. 2:**
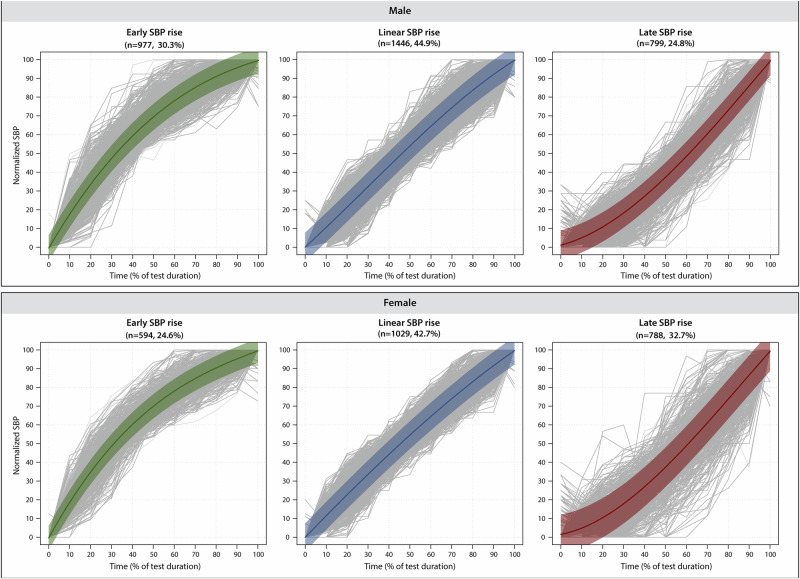
SBP response shapes derived by group-based trajectory modelling (GBTM). GBTM identified three distinct response profiles from the normalized SBP time-series in men and women. The profiles were labelled as an early, linear and late rise in SBP. Each panel shows the individual SBP time-series of the patients assigned to the trajectory (in grey) as well as the trajectory regression line (bold line) and its 90% prediction band (shaded area).

Supplemental Table 1 characterizes the cohort of 5633 patients by sex (mean age, 55.1 years, 43% women). In both men and women, the GBTM algorithm identified three SBP response shapes, representing early, linear and late rises in SBP during graded exercise (Fig. 2*;* Supplemental Fig. 3). Of the three shapes, the linear SBP shape was the most common, comprising 44.9% of the men and 42.7% of the women.

Clinical characteristics per SBP response group are presented for men and women in Table 1. In men, patients with a late rise in SBP had a higher age and BMI and greater prevalence of hypertension, antihypertensive therapy, beta blocker use, diabetes mellitus and CV diseases compared to those with an early or linear rise. In women, no age differences were observed between the SBP response groups, yet the late SBP rise group had higher prevalence of hypertension, antihypertensive therapy and beta blocker use than the other two groups. In both sexes, a late SBP rise was associated with lower peak workload and shorter test duration as well as with higher resting SBP, lower peak SBP and smaller increases in SBP during exercise (*P* ≤ 0.0029 for all comparisons to other SBP response groups) (Table 1, Supplemental Figure 4). These differences in discrete SBP metrics were also seen in individuals without CV risk factors or established disease (*P* ≤ 0.0056 for all comparisons between late and other SBP rises).

**Table 1:** Clinical characteristics by SBP response shapes by sex.

	Men			Women		
	Early SBP rise (n = 977, 30.3%)	Linear SBP rise (n = 1446, 44.9%)	Late SBP rise (n = 799, 24.8%)	Early SBP rise (n = 594, 24.6%)	Linear SBP rise (n = 1029, 42.7%)	Late SBP rise (n = 788, 32.7%)
*Basic clinical data*						
Age, yrs	51.0 ± 15.1	53.0 ± 13.9*	56.6 ± 13.0*†	57.4 ± 12.6	57.3 ± 12.1	57.5 ± 12.8
Weight, kg	86.3 ± 12.4	86.8 ± 13.2	88.5 ± 14.0*†	71.3 ± 11.9	72.4 ± 12.7	73.5 ± 12.8*
Height, cm	179.8 ± 6.5	179.1 ± 6.5*	178.9 ± 6.6*	164.9 ± 5.9	165.4 ± 6.1	165.5 ± 5.7
Body mass index, kg/m²	26.7 ± 3.4	27.0 ± 3.7*	27.6 ± 3.9*†	26.2 ± 4.2	26.5 ± 4.3	26.8 ± 4.4*
*Medical history*						
Hypertension, n (%)	214 (21.9)	361 (25.0)	246 (30.8)*†	137 (23.1)	259 (25.2)	245 (31.1)*†
Treated for hypertension, n (%)	207 (21.2)	347 (24.0)	233 (29.2)*†	134 (22.6)	253 (24.6)	237 (30.1)*†
Use of beta blockers, n (%)	93 (9.5)	234 (16.2)*	218 (27.3)*†	82 (13.8)	170 (16.5)	210 (26.7)*†
Diabetes mellitus, n (%)	47 (4.8)	85 (5.9)	76 (9.5)*†	27 (4.6)	48 (4.7)	30 (3.8)
Treated for diabetes, n (%)	41 (4.2)	76 (5.3)	71 (8.9)*†	24 (4.0)	41 (4.0)	24 (3.1)
Lipid-lowering drugs, n (%)	135 (13.8)	217 (15.0)	163 (20.4)*†	71 (12.0)	134 (13.0)	99 (12.6)
Anticoagulants, n (%)	101 (10.3)	231 (16.0)*	196 (24.5)*†	74 (12.5)	121 (11.8)	125 (15.9)†
Heart failure, n (%)	2 (0.2)	9 (0.6)	6 (0.8)	1 (0.2)	2 (0.2)	3 (0.4)
Atrial fibrillation, n (%)	16 (1.6)	46 (3.2)*	41 (5.1)*†	6 (1.0	20 (1.9)	20 (2.5)*
IHD before test, n (%)	68 (7.0)	163 (11.3)*	144 (18.0)*†	63 (10.6)	105 (10.2)	87 (11.0)
Cardiac disease, n (%)	86 (8.8)	212 (14.7)*	174 (21.8)*†	66 (11.1)	123 (12.0)	106 (13.5)
Cerebrovascular disease, n (%)	15 (1.5)	16 (1.1)	9 (1.1)	4 (0.7)	9 (0.9)	4 (0.5)
Cardiovascular disease, n (%)	98 (10.0)	224 (15.5)*	178 (22.3)*†	70 (11.8)	131 (12.7)	109 (13.8)
*Graded exercise test data*						
Resting systolic BP, mmHg	128.3 ± 17.9	128.7 ± 18.1	135.8 ± 19.2*†	128.0 ± 21.2	130.1 ± 19.8*	135.7 ± 21.1*†
Resting heart rate, bpm	81.4 ± 14.0	80.1 ± 14.1*	77.4 ± 14.2*†	83.0 ± 13.3	82.0 ± 13.5	78.7 ± 13.1*†
Peak systolic BP, mmHg	205.8 ± 23.0	204.7 ± 23.2	199.4 ± 24.7*†	194.1 ± 24.0	194.0 ± 23.5	190.2 ± 24.9*†
Peak heart rate, bpm	166.7 ± 19.4	160.2 ± 19.2*	152.0 ± 21.9*†	159.3 ± 17.7	156.6 ± 18.5*	151.4 ± 19.1*†
Test duration, min	10.8 ± 2.3	10.5 ± 2.2*	10.0 ± 2.3*†	10.4 ± 2.1	10.4 ± 2.4	10.2 ± 2.3†
Peak RPE, score	17.6 ± 0.9	17.7 ± 0.9	17.6 ± 0.9	17.4 ± 0.8	17.5 ± 0.8	17.4 ± 0.8
Peak workload, Watts	229.5 ± 50.7	214.8 ± 42.5*	202.0 ± 38.7*†	138.5 ± 27.4	136.6 ± 26.5	133.6 ± 25.0*†
Percentage of predicted workload, %	96.9 ± 14.7	93.4 ± 14.3*	91.0 ± 13.9*†	100.2 ± 15.0	98.0 ± 14.4*	96.2 ± 14.4*†

Values are mean ± SD or count (%).Cardiac disease included IHD, heart failure, cardiomyopathy and arrhythmia requiring clinical intervention. Cardiovascular disease additionally included stroke.

*<0.05 versus early rise, †<0.05 versus linear rise.

*BP* blood pressure, *bpm* beats per minute, *IHD* ischaemic heart disease, *RPE* rate of perceived exertion.

### Clinical correlates of SBP response groups

In multinomial logistic regression, a late rise in SBP was independently associated with older age, lower heart rate, higher BMI, diabetes mellitus, cardiac disease, beta blocker use, absence of lipid-lowering drugs and lower ppWL in men (Table 2). In women, a late rise was also associated with lower heart rate, higher BMI, beta blocker use and lower exercise capacity (Table 2). Among individuals without CV risk factors or established disease, late rise in SBP was independently related to age, heart rate, BMI and ppWL in men and to heart rate and ppWL in women (Supplemental Table 2).

**Table 2:** Multivariable-adjusted clinical correlates of linear and late rise in SBP in multinomial logistic regression by sex.

Covariable	Linear SBP rise		Late SBP rise	
	Odds ratio (95% CI)	*P* value	Odds ratio (95% CI)	*P* value
*Men*				
Age, per +10 years	1.09 (1.02 to 1.16)	0.0082	1.29 (1.20 to 1.40)	<0.0001
Heart rate at rest, +10 bpm	0.93 (0.87 to 0.98)	0.013	0.82 (0.76 to 0.88)	<0.0001
Body mass index, per +5 kg/m²	1.13 (1.01 to 1.28)	0.038	1.35 (1.17 to 1.55)	<0.0001
Diabetes mellitus	1.11 (0.74 to 1.66)	0.59	1.54 (1.00 to 2.36)	0.049
Cardiac disease	1.36 (0.99 to 1.87)	0.057	1.46 (1.04 to 2.07)	0.031
Beta blockers	1.42 (1.05 to 1.91)	0.023	2.06 (1.49 to 2.85)	<0.0001
Lipid-lowering drugs	0.67 (0.49 to 0.90)	0.0087	0.59 (0.42 to 0.83)	0.0022
Percentage predicted workload, per +10%	0.83 (0.78 to 0.88)	<0.0001	0.72 (0.67 t 0.77)	<0.0001
*Women*				
Heart rate at rest, +10 bpm	0.93 (0.86 to 1.01)	0.062	0.77 (0.71 to 0.84)	<0.0001
BMI, per +5 kg/m²	1.06 (0.94 to 1.20)	0.90	1.17 (1.03 to 1.34)	0.017
Beta blockers	1.13 (0.82 to 1.56)	0.47	1.72 (1.24 t 2.38)	0.0011
Percentage predicted workload, per +10%	0.89 (0.83 to 0.96)	0.0021	0.80 (0.74 to 0.87)	<0.0001

Odds ratios represent the multivariable-adjusted risk for the SBP response shape relative to the risk of presenting an early rise in SBP during graded exercise. Covariables significant for either a linear or a late rise in SBP are shown. Covariables considered as predictors included age, BMI, resting heart rate, hypertension, diabetes mellitus, renal failure, COPD, cardiac disease, the use of antihypertensive medication, beta blockers, lipid-lowering drugs, and anticoagulant drugs, and the percentage predicted workload (reflecting exercise capacity).

### SBP shape trajectories and all-cause mortality

During a median follow-up of 8.3 years (5-95^th^ percentile, 3.6-14.0 years), encompassing 47,993 person-years at risk, 296 patients (5.3%) died. Supplemental Table 3 lists the event rates by SBP response group and sex.

**Fig. 3:**
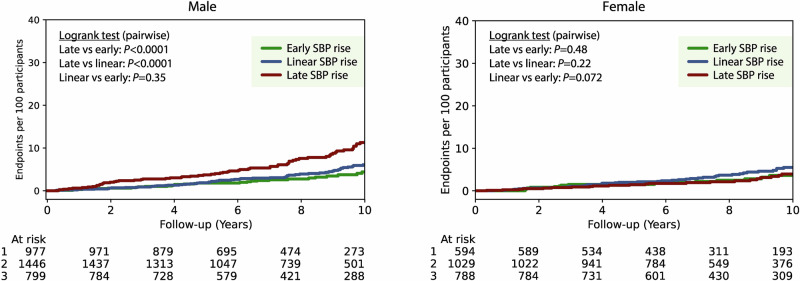
Kaplan-Meier for unadjusted incidence of all-cause mortality across the shape-based SBP response groups by sex. Incidence of death was compared between SBP response groups using pairwise Logrank tests for male (left) and female (right) participants.

Figure 3 illustrates the cumulative incidence of all-cause mortality by SBP response group and sex. In men, unadjusted analyses showed a significantly higher mortality rate in the late SBP rise group as compared to the early or linear rise groups (*P*_LOG-RANK_ < 0.0001 for both). After full adjustment, men with a late rise in SBP had a 66% higher risk of all-cause mortality than those with an early SBP rise (adjusted HR: 1.66 [95% CI: 1.10 to 2.50], *P* = 0.015) (Table 3). In contrast, no significant associations were observed between SBP response group and mortality in women (e.g. adjusted HR for late vs. early rise: 1.06 [0.60 to 1.90], *P* = 0.84) (Fig. 3 and Table 3).

**Table 3:** Cox regression models for all-cause mortality across SBP response groups.

SBP response group	Unadjusted HR (95% CI)	*P* value	Adjusted HR (95% CI)	*P* value
*Men*				
Early rise	*reference*		*reference*	
Linear rise	1.21 (0.82 to 1.80)	0.34	1.18 (0.79 to 1.76)	0.43
Late rise	2.31 (1.56 to 3.41)	<0.0001	1.66 (1.10 to 2.50)	0.015
*Women*				
Early rise	*reference*		*reference*	
Linear rise	1.60 (0.95 to 2.69)	0.078	1.47 (0.87 to 2.49)	0.15
Late rise	1.22 (0.70 to 2.15)	0.48	1.06 (0.60 to 1.90)	0.84

Hazard ratios express the risk for all-cause mortality within the SBP response shape relative to people with an early rise in SBP. Adjusted HR accounted for differences in age, BMI, resting SBP and heart rate, diabetes mellitus, renal failure, COPD, cardiac disease, antihypertensive drugs, beta blockers, lipid-lowering drugs, anticoagulant drugs and percentage predicted workload.

## Discussion

In this study, we applied trajectory modelling to unravel the physiological appearance and clinical meaning of the SBP response shape during graded exercise. In a large patient cohort referred for cycle ergometry, our GBTM-based approach identified three distinct SBP response shapes in both men and women: an early rise/levelling off shape, a linear rise and a late rise. A late rise in SBP was associated with worse CV risk profile, as indicated by higher age, obesity, beta blocker use and lower exercise capacity, and with a more unfavorable profile of traditional SBP exercise metrics. In men, a late SBP rise predicted all-cause mortality, independently from established CV risk markers. Overall, SBP shape assessment may provide a more nuanced understanding of the SBP response during exercise and have clinical implications for CV risk stratification.

### Algorithmic assessment of the SBP response shape

Previous studies on the SBP response during exercise have primarily focused on discrete metrics such as peak SBP [10, 19] and two-point SBP slopes [11, 20]. While these metrics have prognostic value, they may oversimplify the complex dynamics of SBP during exercise. A recent study by Carlén et al. assessed the predictive value of different SBP responses at the end of exercise, concluding that the change in SBP between the last two measurements (standardized for increase in workload) does not independently predict incident CV disease, except if SBP drops [12]. However, like conventional SBP exercise metrics, these end-exercise changes still convert a continuous physiological response into a measurable yet simplified metric, while imposing artificial thresholds on (part of) the raw SBP curve. Such discrete metrics rely on hard cutoffs for classification that may introduce bias and fail to capture the SBP trajectory throughout the exercise test. By contrast, an integrative analysis of the entire SBP response curve – using an unbiased, algorithmic approach – can overcome these limitations and provide a more comprehensive assessment of SBP dynamics during exercises.

This is the first study to algorithmically assess the (non-)linearity of the SBP response in patients with an adequate rise in SBP. Our approach extracts the SBP shape directly from the raw time-series data and classifies it as an early, linear or late rise in SBP. Based on our findings, trajectory-based assessment of the SBP shape may offer additional insights into vascular health and long-term prognosis.

In this study, graded exercise protocols were considered, during which time and workload change proportionally every minute. As such, they could be used interchangeably when extracting the SBP response shape. Future studies, however, may need to correct for workload instead of test time if a stepwise exercise protocols with less frequent increments in workload are applied and/or if absolute SBP levels or SBP slopes are investigated.

### Physiological and clinical correlates of the SBP response shape

The SBP shape seems to capture key information from the raw SBP time-series, as reflected in its relationship with discrete SBP metrics. In both sexes, a late SBP rise was associated with unfavorable deviations in these metrics, including elevated resting SBP and attenuated peak SBP – both which have been linked to all-cause mortality [21, 22]. Furthermore, SBP response shape appears closely related to exercise capacity, supporting the close entanglement between cardiorespiratory fitness and SBP dynamics [20, 23]. Further investigation is needed to determine whether the SBP shape provides unique clinical value beyond the established exercise metrics.

Besides these “inherent” physiological correlates, a late rise in SBP was linked to an unfavorable CV risk profile. In men, it strongly correlated with older age, obesity, diabetes mellitus, established heart disease, the use of beta blockers and lower exercise capacity (expressed as ppWL), while in women associations were observed with obesity, beta blockers and lower exercise capacity. Prior studies have already documented the impact of ageing [15, 23], cardiometabolic disturbances [24, 25] and established heart disease [26] on discrete SBP exercise metrics. Beta blockers are also known to blunt the SBP response to exercise [1], while cardiorespiratory fitness relates directly to peak SBP [20, 23]. Our findings suggest that a late rise in SBP may serve as a marker of CV risk and (sub)clinical CV dysfunction.

Prospective validation studies are warranted to confirm the identified clinical factors and determine whether these factors act as confounders, mediators or moderators in the SBP shape response to exercise. Additionally, future research should explore how mechanic and molecular mechanisms that regulate the SBP response to exercise – such as cardiac output, sympathetic activity, vascular resistance and thermoregulation – determine the shape of that response [2].

### The predictive value of the SBP response shape

Unlike flattening of surrogate measures of stroke volume [27, 28], a flattened SBP response at the end of graded exercise testing was not associated with future adverse events [12]. In our study, the SBP response shape independently predicted all-cause mortality, but only in men. Specifically, a late rise in SBP was associated with increased mortality in men but not in women. This sex difference may originate from physiological differences in vascular function, hormonal influences, or differences in CV risk factor profiles. In men, the link between a late rising shape and increased mortality may be driven by its strong relation with age, obesity, diabetes mellitus and impaired exercise capacity as well as with discrete SBP metrics linked to all-cause mortality [22]. Future studies should validate the predictive value of SBP response shapes and dig deeper into the potential sex discrepancy in this association.

#### Perspectives

Our GBTM-based approach enables a qualitative assessment and unbiased interpretation of the shape of the SBP response during graded exercise. This integrative approach may pave the way to a standardized framework for objective evaluation of the SBP response shape in both research and clinic, accelerating investigations on the utility of integrating SBP response shape analysis into exercise testing protocols. Prospective studies should determine if SBP shape characterization offers additive value for disease prediction, diagnosis and prognosis beyond conventional markers. Additionally, exploring the impact of interventions such as antihypertensive therapy or exercise training on the SBP response shape may provide insights into novel strategies for CV risk management. If validated in future studies, algorithmic SBP shape classifications could be integrated into routine exercise testing to refine CV risk assessment and guide clinical decision-making.

#### Strengths and limitations

The strengths of our study include the use of a large, well-characterized patient cohort, a robust statistical approach for the SBP shape modelling, and long follow-up of mortality. We employed an objective, algorithmic method to classify the SBP response shapes, minimizing subjectivity in data interpretation. However, we acknowledge several methodological caveats related to SBP shape assessment. First, maximal clinical exercise tests with tailored ramp protocols were analyzed. Consistent with how cardiorespiratory responses are compared in clinical settings, we thus compared SBP response shapes at relative and not absolute exercise intensities. Our findings may not directly translate to other exercise test protocols. Second, the variation in timing of SBP measurements may have affected the precision of the SBP shape assessment. Although challenging, future studies should investigate whether tighter measurement protocols influence the reproducibility of SBP response shapes. Third, SBP was measured using a Doppler probe. Although this is a practical and valid method used in clinical exercise testing, formal reproducibility data during dynamic exercise is lacking, and the concordance with manual and automated auscultation for SBP shape assessment remains to be determined. Fourth, a minimum of four SBP measurements during exercise is required to distinguish a non-linear from a linear shape, which limits applicability in individuals with fewer measurements. Fifth, our analysis focused exclusively on SBP risers (≥30 mmHg rise in SBP) for two reasons: (1) other response patterns such as an SBP drop and overall low SBP increase are visually identifiable and already well-documented as adverse, and (2) non-rising shapes may present challenges for time-series processing (e.g. 0-1 normalization of SBP is not feasible for a completely flat response) and for precise identification of their trajectory due to their low occurrence. Additionally, the retrospective nature of the study does not allow to infer causality. Our cohort consisted of patients referred for clinical exercise testing, potentially leading to an underrepresentation of healthy individuals. This may limit the generalizability of our findings to asymptomatic individuals. Future prospective studies on ethnically diverse populations, along with mechanistic investigations, are needed to validate and expand on our findings.

#### Conclusions

We presented a novel algorithmic approach for objective assessment of the SBP shape during graded exercise, which identified three distinct SBP response shapes. A late rise in SBP was associated with worse CV risk profile and more unfavorable conventional SBP exercise metrics. A late SBP rise predicted all-cause mortality in men, independent from established CV risk markers. Our findings advocate future studies on the mechanisms driving the SBP response shape and on the added value of SBP shape assessment for patient risk stratification.

## Summary

### What is known about the topic?

- Systolic blood pressure (SBP) normally increases linearly with exercise intensity, while a drop or exaggerated SBP response are linked to adverse cardiovascular outcomes.
- Previous research investigated discrete SBP markers like peak values or linear slopes, which may oversimplify SBP dynamics and not sufficiently capture the way in which SBP evolves during exercise.
- Trajectory modeling techniques could help objectively characterize SBP responses to exercise based in an integrative way, potentially revealing clinically significant subgroups and their associations with health outcomes.

### What does this study add?

- We explored the physiological and clinical relevance of the SBP response shape to exercise.
- Using trajectory modelling, three distinct SBP response shapes were identified – an early, linear, and late rise. A late rise in SBP was associated with an unfavorable cardiovascular risk profile, including lower exercise capacity, and independently predicted all-cause mortality in men.
- Beyond traditional SBP metrics, characterizing the SBP response shape may enhance cardiovascular risk assessment in clinical exercise testing.

## Supplementary information

Supplemental tables and figures

## Data Availability

The data underlying this article are available from the corresponding author (K.H.) upon reasonable request.
