# Supplementary material for: The shape of the systolic blood pressure response during graded exercise: methodology, correlates and predictive value
Source: J Hum Hypertens. 2025 Nov 14;40(1):37–44. doi: 10.1038/s41371-025-01093-7 (PMC12807865; doi:10.1038/s41371-025-01093-7)
Supplement: Supplementary file 1 — Supplemental tables and figures [file 41371_2025_1093_MOESM1_ESM.docx]

**The Shape of the Systolic Blood Pressure Response during Graded Exercise:
Methodology, Correlates and Predictive Value**

Nicholas Cauwenberghs, Anna Carlén, Thomas Lindow, Viktor Elmberg, Lars Brudin,
Magnus Ekström, Kristofer Hedman

*Supplemental data*

**Supplemental Table 1. Clinical characteristics by sex.**

|  | **Female (n=2411)** | **Male (n=3222)** | ***P* value** |
| --- | --- | --- | --- |
| *Basic clinical data* |  |  |  |
| Age, yrs | 57.4 ± 12.5 | 53.3 ± 14.2* | <0.0001 |
| Weight, kg | 72.5 ± 12.6 | 87.0 ± 13.2* | <0.0001 |
| Length, cm | 165.3 ± 5.9 | 179.3 ± 6.5* | <0.0001 |
| Body mass index, kg/m² | 26.5 ± 4.3 | 27.1 ± 3.7* | <0.0001 |
| *Medical history* |  |  |  |
| Hypertension, n (%) | 641 (26.6) | 821 (25.5) | 0.35 |
| Treated for hypertension, n (%) | 624 (25.9) | 787 (24.4) | 0.21 |
| Use of beta blockers, n (%) | 462 (19.2) | 545 (16.9)* | 0.029 |
| Diabetes mellitus, n (%) | 105 (4.4) | 208 (6.7)* | 0.0007 |
| Treated for diabetes, n (%) | 89 (3.7) | 188 (5.8)* | 0.0002 |
| Lipid-lowering drugs, n (%) | 304 (12.6) | 515 (16.0)* | 0.0004 |
| Anticoagulants, n (%) | 320 (13.3) | 528 (16.4)* | 0.0012 |
| Heart failure, n (%) | 6 (0.3) | 17 (0.5) | 0.10 |
| Atrial fibrillation, n (%) | 46 (1.9) | 103 (3.2)* | 0.0029 |
| IHD before test, n (%) | 255 (10.6) | 375 (11.6) | 0.21 |
| Cardiac disease, n (%) | 295 (12.2) | 472 (14.7)* | 0.0090 |
| Cerebrovascular disease, n (%) | 17 (0.7) | 40 (1.3)* | 0.047 |
| Cardiovascular disease, n (%) | 310 (12.9) | 500 (15.5)* | 0.0049 |
| *Graded exercise test data* |  |  |  |
| Resting systolic BP, mmHg | 131.4 ± 20.8 | 130.3 ± 18.6* | 0.041 |
| Resting heart rate, bpm | 81.2 ± 13.4 | 79.9 ± 14.2* | 0.0005 |
| Peak systolic BP, mmHg | 192.8 ± 24.1 | 203.7 ± 23.6* | <0.0001 |
| Peak heart rate, bpm | 155.6 ± 18.7 | 160.1 ± 20.7* | <0.0001 |
| Test duration, min | 10.3 ± 2.3 | 10.5 ± 2.3* | <0.0001 |
| Peak RPE, score | 17.4 ± 0.8 | 17.6 ± 0.9* | <0.0001 |
| Peak workload, watts | 136.1 ± 26.3 | 215.9 ± 45.5* | <0.0001 |
| Percentage of predicted workload, % | 98.0 ± 14.6 | 93.9 ± 14.5* | <0.0001 |
| Values are mean±SD or count (%). Abbreviations: BP, blood pressure; IHD, ischaemic heart disease; RPE, rate of perceived exertion. | | |  |

**Supplemental Table 2. Multivariable-adjusted clinical correlates of linear and late rise in SBP in multinomial logistic regression in people free from cardiovascular risk factors and established disease.**

| **Covariable** | **Linear rise pattern** | | **Late rise pattern** | |
| --- | --- | --- | --- | --- |
|  | Odds ratio (95% CI) | *P* value | Odds ratio (95% CI) | *P* value |
| *Men* |  |  |  |  |
| Age, per +10 years | 1.12 (1.04 to 1.21) | 0.0040 | 1.33 (1.20 to 1.47) | <0.0001 |
| Heart rate at rest, +10 bpm | 0.97 (0.89 to 1.05) | 0.42 | 0.81 (0.73 to 0.91) | 0.0003 |
| BMI, per +5 kg/m² | 1.18 (0.94 to 1.48) | 0.16 | 1.41 (1.05 to 1.90) | 0.021 |
| Percentage predicted workload, per +10% | 0.83 (0.77 to 0.89) | <0.0001 | 0.71 (0.64 to 0.78) | <0.0001 |
| *Women* |  |  |  |  |
| Heart rate at rest, +10 bpm | 0.88 (0.79 to 0.99) | 0.026 | 0.77 (0.68 to 0.87) | <0.0001 |
| Percentage predicted workload, per +10% | 0.86 (0.78 to 0.95) | 0.0028 | 0.73 (0.66 to 0.82) | <0.0001 |
| Models were run on a healthy reference group of people free from traditional CV risk factors (i.e. hypertension, diabetes mellitus, and obesity) and cardiovascular disease, COPD and renal failure. Odds ratios represent the multivariable-adjusted risk for the SBP response group relative to the risk of presenting an early rise in SBP during graded exercise. Covariables significant for either a linear or a late rise in SBP are shown. Covariables considered as predictors included age, BMI, resting heart rate, lipid-lowering drugs, and anticoagulant drugs, and the percentage predicted workload (reflecting exercise capacity). | | | | |

**Supplemental Table 3. All-cause mortality across SBP response group and sex.**

|  | ***Men*** | | | ***Women*** | | |
| --- | --- | --- | --- | --- | --- | --- |
|  | **Early rise (n=977)** | **Linear rise (n=1446)** | **Late rise (n=799)** | **Early rise (n=564)** | **Linear rise (n=1029)** | **Late rise (n=788)** |
| Number of events | 37 | 72 | 78 | 19 | 56 | 34 |
| Event rate (per 1000 person-years) | 4.6 | 5.9 | 11.3 | 3.8 | 6.3 | 4.9 |
| The median follow-up time and 5-95% percentile was 8.0 (3.6 to 14.0) years in men and 8.5 (3.6 to 13.9) years in women. | | | | | | |

**
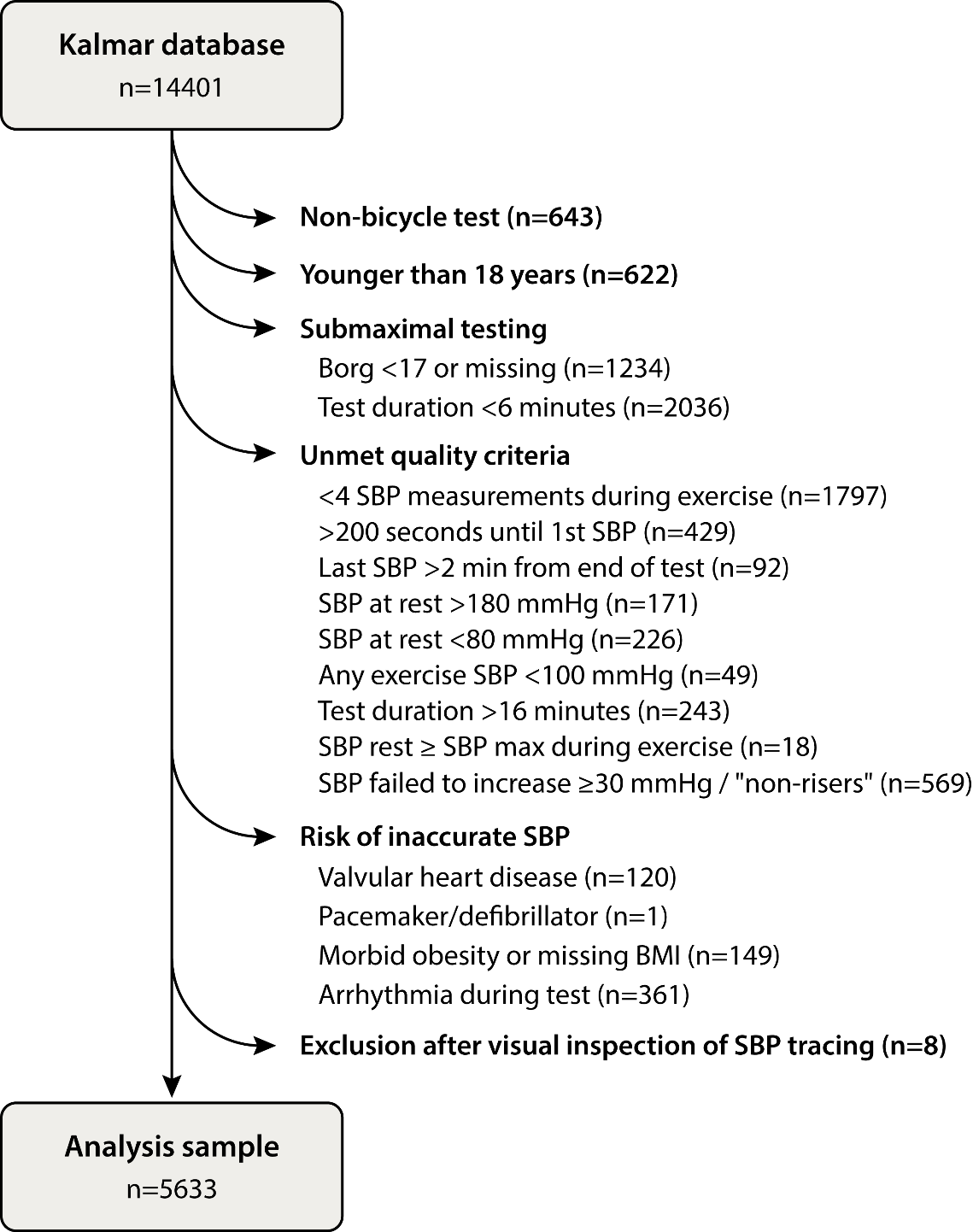
Supplemental Figure 1. Flow chart.**

**
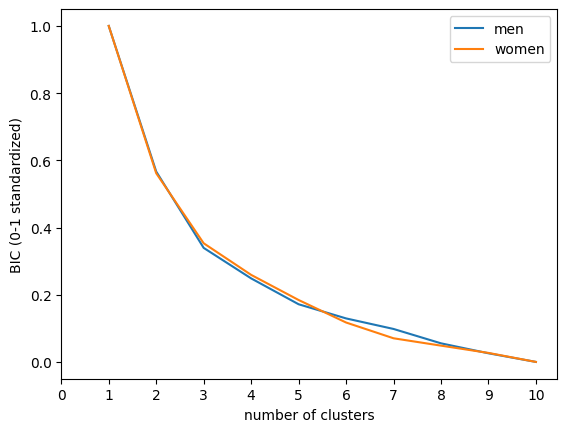
Supplemental Figure 2. BIC elbow plot to determine optimal number of SBP response shapes.** In both men and women, the preferred number of trajectories was three, as indicated by the “elbow-like” hinge point on the curves. We thus derived three trajectories in both sexes.

**
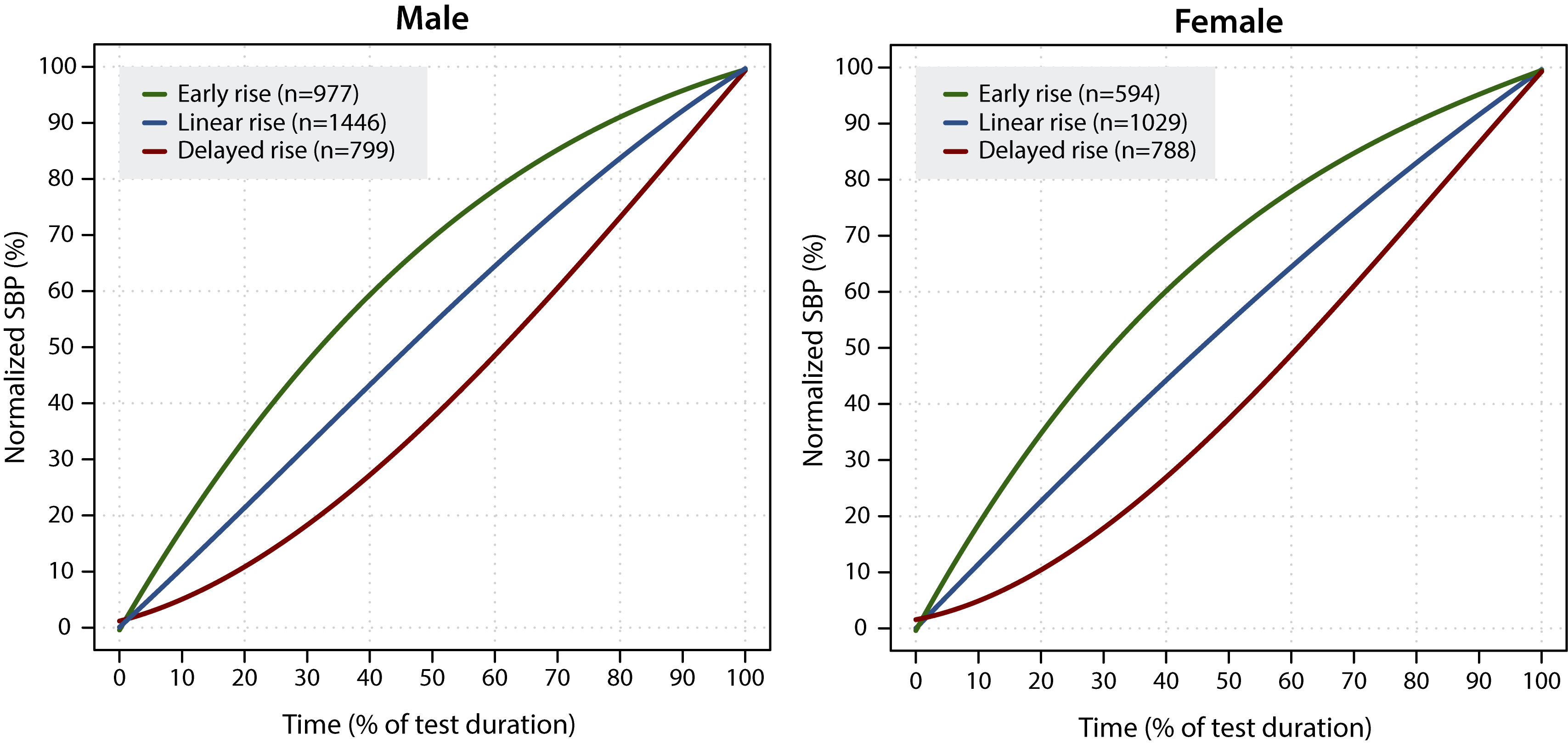
Supplemental Figure 3. Sex-specific SBP response shapes as identified by group-based trajectory modelling (GBTM).** In both men and women, the GBTM algorithm identified three SBP response shapes, representing an early, linear and late rise in SBP during graded exercise.


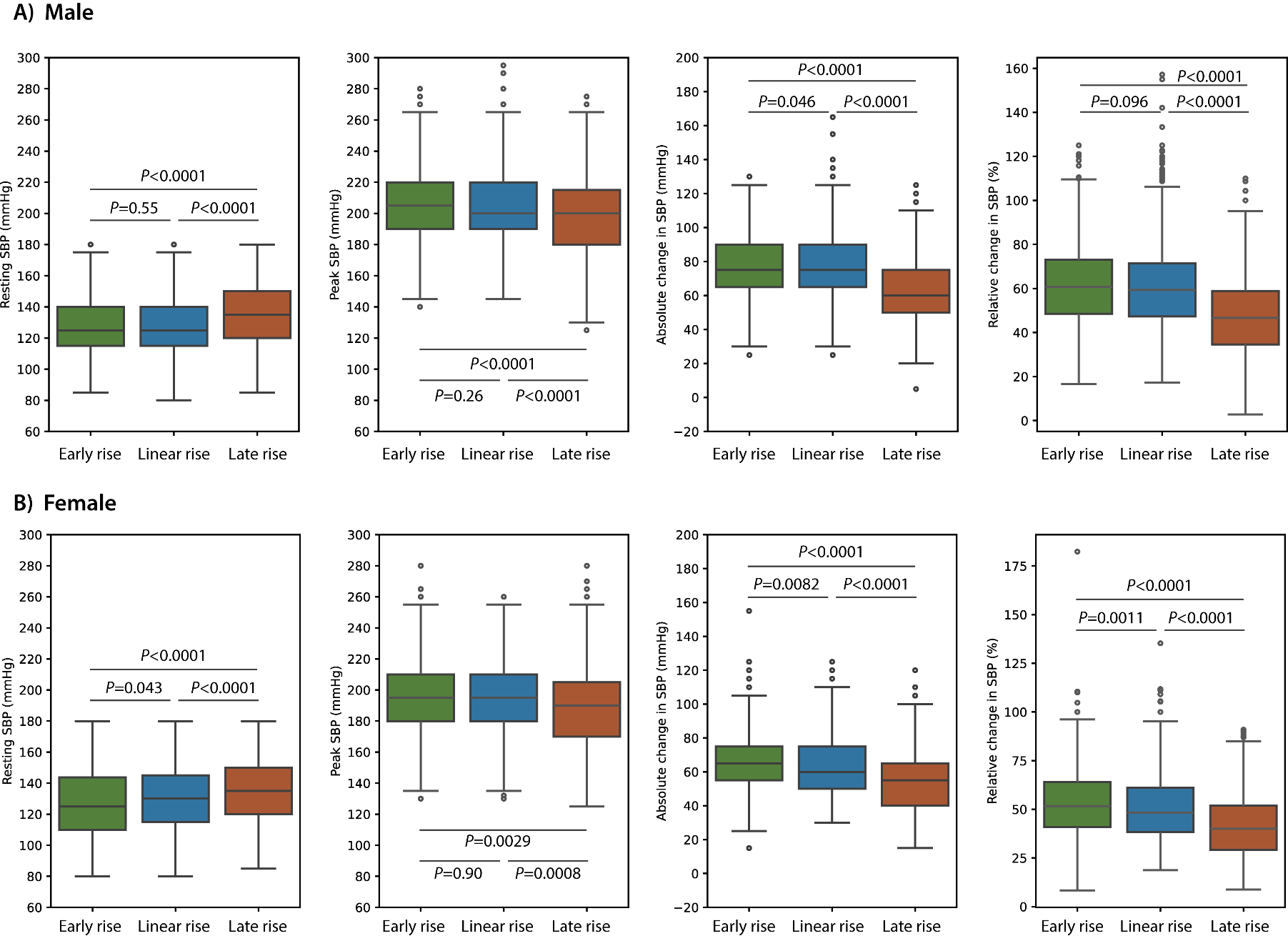
**Supplemental Figure 4. Discrete SBP metrics across SBP response groups for male (A) and female (B) patients.**
